# Differential Sensitivity of ERBB2 Kinase Domain Mutations towards Lapatinib

**DOI:** 10.1371/journal.pone.0026760

**Published:** 2011-10-28

**Authors:** Rama Krishna Kancha, Nikolas von Bubnoff, Natalie Bartosch, Christian Peschel, Richard A. Engh, Justus Duyster

**Affiliations:** 1 Department of Internal Medicine III, Technical University of Munich, Munich, Germany; 2 NORSTRUCT, Department of Chemistry, University of Tromsø, Tromsø, Norway; University of Chicago, United States of America

## Abstract

**Background:**

Overexpression of the ERBB2 kinase is observed in about one-third of breast cancer patients and the dual ERBB1/ERBB2 kinase inhibitor lapatinib was recently approved for the treatment of advanced ERBB2-positive breast cancer. Mutations in the ERBB2 receptor have recently been reported in breast cancer at diagnosis and also in gastric, colorectal and lung cancer. These mutations may have an impact on the clinical responses achieved with lapatinib in breast cancer and may also have a potential impact on the use of lapatinib in other solid cancers. However, the sensitivity of lapatinib towards clinically observed ERBB2 mutations is not known.

**Methodology/Principal Findings:**

We cloned a panel of 8 clinically observed ERBB2 mutations, established stable cell lines and characterized their sensitivity towards lapatinib and alternative ERBB2 inhibitors. Both lapatinib-sensitive and lapatinib-resistant ERBB2 mutations were observed. Interestingly, we were able to generate lapatinib resistance mutations in wt-ERBB2 cells incubated with lapatinib for prolonged periods of time. This indicates that these resistance mutations may also cause secondary resistance in lapatinib-treated patients. Lapatinib-resistant ERBB2 mutations were found to be highly resistant towards AEE788 treatment but remained sensitive towards the dual irreversible inhibitors CL-387785 and WZ-4002.

**Conclusions/Significance:**

Patients harbouring certain ERBB2 kinase domain mutations at diagnosis may not benefit from lapatinib treatment. Moreover, secondary lapatinib resistance may develop due to kinase domain mutations. Irreversible ERBB2 inhibitors may offer alternative treatment options for breast cancer and other solid tumor patients harbouring lapatinib resistance mutations. In addition, these inhibitors may be of interest in the scenario of secondary lapatinib resistance.

## Introduction

ERBB2 amplification or overexpression was reported in 30% of breast cancers and is correlated with poor prognosis, increased metastatic potential and resistance to apoptosis[1]. More recently, mutations in the ERBB2 kinase domain were also reported in various solid cancers[2]–[7]. Previous studies have shown that a solid tumor entity can be uniformly addicted to a specific oncogenic kinase, and the presence of activating mutations within a specific kinase determines response to therapeutic kinase inhibition. For example, activating ErbB1 mutations determine the response to EGFR kinase inhibitors such as gefitinib and erlotinib[8]. Moreover, it has been shown that the specific type of mutation within the kinase domain of an oncoprotein determines differential responses towards different kinase inhibitors[9], [10]. Thus, it is important to biochemically characterize individual mutations and to devise experimental cellular systems to test the efficacy of inhibitors against them. A comprehensive study to establish drug sensitivity profiles for mutations reported in the clinic allows selection of the appropriate treatment strategy in patients. To this end, we aimed to establish drug sensitivity profiles of ERBB2 kinase domain mutants against ERBB2 inhibitors.

Lapatinib is a dual inhibitor of EGFR and ERBB2 kinases. In the present study the efficacy of lapatinib against ERBB2 variants was studied. Moreover, a cell based screening strategy was employed to identify lapatinib resistant ERBB2 kinase domain mutations. The effect of another reversible dual EGFR/ERBB2 inhibitor AEE 788, was tested against ERBB2 mutants. Together, comprehensive drug sensitivity profiles for various ERBB2 mutations that were reported in several cancers were established along with the identification of lapatinib resistant mutations. Furthermore, irreversible ERBB2 inhibitors were identified which potentially can overcome lapatinib resistance.

## Results and Discussion

### Identification of lapatinib resistant ERBB2 kinase domain mutations

**Figure 1:**
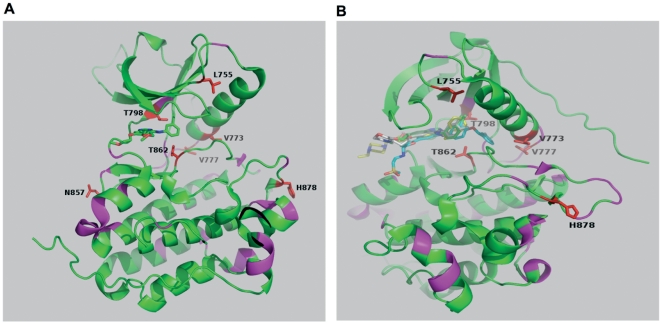
Schematic representation of ERBB2 mutations analyzed. (**A and B**) The side chains of mutants considered in this study are plotted (red sticks) together with a schematic representation of the protein fold using the crystal structure of EGFR kinase in complex with erlotinib (green sticks). B) is a view roughly orthogonal to A) and shows additional inhibitors gefitinib (yellow sticks) and lapatinib (blue sticks) superimposed at the ATP binding site.

It has been demonstrated that the drug sensitivity of different mutations varies against selective inhibitors. Thus, we aimed to test the efficacy of reversible ERBB2 inhibitors lapatinib and AEE788 against a panel of ERBB2 kinase domain mutations that were reported in various solid cancers (Table 1). Analogous mutations in EGFR were reported for most of the ERBB2 mutations analyzed in this study (Table S1), suggesting that these mutations are not passenger mutations but functionally important. Additionally, a gatekeeper mutation T798M was cloned for analysis. ERBB2-T798M is analogous to EGFR-T790M that was shown to cause resistance towards EGFR inhibitors[10], [11]. The locations of the kinase domain mutants investigated in this study are depicted in Figure 1. Four mutations are located in the N-lobe of the kinase. L755 is located at a loop adjacent to helix C, V773 and V777 are at or near the C-terminal portion of helix C, and T798 is at the gatekeeper position in the ATP binding site (Figure 1A and B). Of the remainder, N857 is located in helix D, T862A forms the base of the ATP binding site, and H878 is in the activation loop.

**Table 1:** Summary of ERBB2 mutants analyzed along with the IC50 values against reversible inhibitors lapatinib and AEE788.

ERBB2 mutation	Exon	Functional region	Cancer type	Lapatinib	AEE788	Reference
WT	NA	NA	Breast cancer	30	257	NA
L755S	19	ATP binding region	Breast and gastric cancer	>2000	897	4
L755P	19	ATP binding region	NSCLC	1545	1216	2,3
V773A	20	ATP binding region	SCCHN	146	200	6
V777L	20	ATP binding region	Gastric, colon and lung	27	215	3,4
T798M	20	Gate keeper residue	NA	1433	>2000	NA
N857S	21	Activation loop	Ovarian cancer	75	246	2
T862A	21	Activation loop	Primary gastric cancer	125	191	7
H878Y	21	Activation loop	Hepatocellular carcinoma	14	168	5

ERBB2 kinase domain mutations that were reported in solid cancers were shown along with their structural position and IC50 values against lapatinib and AEE 788. IC50 values were calculated based on Figures 1C and 1D.

**Figure 2:**
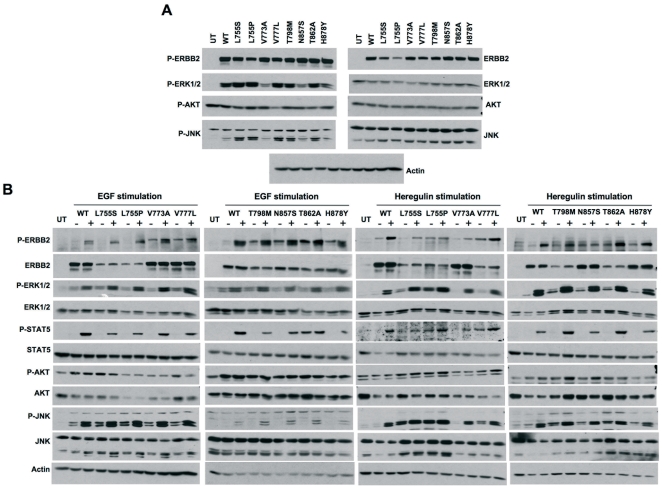
Biochemical analysis of ERBB2 mutants. (A) HEK293 cells were transfected with either wild type (WT) or mutant ERBB2 for 36 hours and analyzed for autophosphorylation and activation of downstream signaling molecules. Untransfected (UT) cells were taken as control for ERBB2 expression. (B) HEK293 cells were transfected with a combination of ERBB2 (WT or mutant) and EGFR (left two panels) or ERBB3 constructs (right two panels) for 36 hours followed by serum starvation for 12 hours. Cells were then stimulated with either EGF (left two panels) or heregulin (right two panlels) for 5 minutes and analyzed for the activation of ERBB2 as well as downstream signaling pathways by western blotting. Untransfected (UT) cells were used as control.

**Figure 3:**
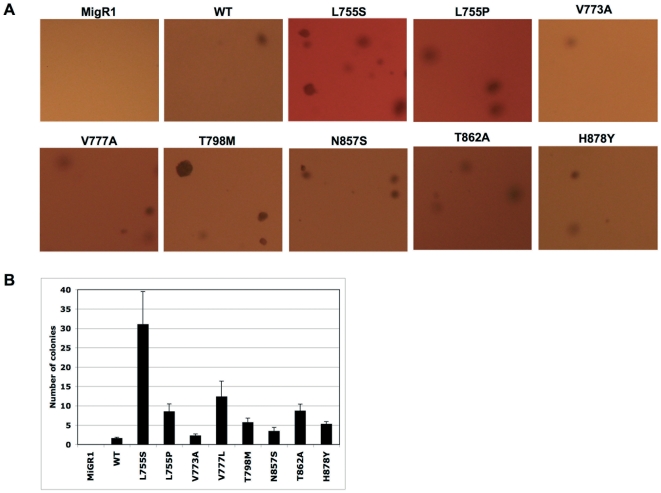
Anchorage-independent growth of ERBB2 mutants.

All the mutations analyzed retained autokinase activity and activated downstream signaling pathways when expressed in HEK293 cells (Figure 2A). Moreover mutations L755S, L755P, V777L, T798M and T862A displayed enhanced activation of JNK/SAPK and to a lesser extent of ERK1/2 compared to wt-ERBB2 (Figure 2A). Enhanced autophosphorylation as well as activation of downstream signaling molecules was also observed upon stimulation with either EGF or heregulin of serum starved HEK293 cells expressing ERBB2 in combination with EGFR or ERBB3 (Figure 2B) indicating that the mutations did not interfere with ligand-induced heterodimerization of the ERBB2 mutants with EGFR or ERBB3. Early passage NMuMg cells (a non-transformed mouse mammary epithelial cell line) stably expressing wt- or mutant-ERBB2 formed distinct colonies in six-well cell culture plates (Figure S1) as well as in soft agar (Figure 3A). Hereby, ERBB2-L755S, ERBB2-L755P, ERBB2-V777L and ERBB2-T862A formed more colonies compared to wt- ERBB2 (Figure 3B) indicating an enhanced transforming potential. Interestingly, late passage NMuMg cells stably expressing ERBB2-L755S, ERBB2-L755P, ERBB2-V777L, ERBB2-T798M, ERBB2-T862A and ERBB2-H878Y also formed colonies in liquid culture in contrast to wt-ERBB2 also supporting enhanced transforming potential of these ERBB2 mutants (Figure S2). Similar observations were made in a recent report with NIH3T3 cells expressing ERBB2-L755S[12].

**Figure 4:**
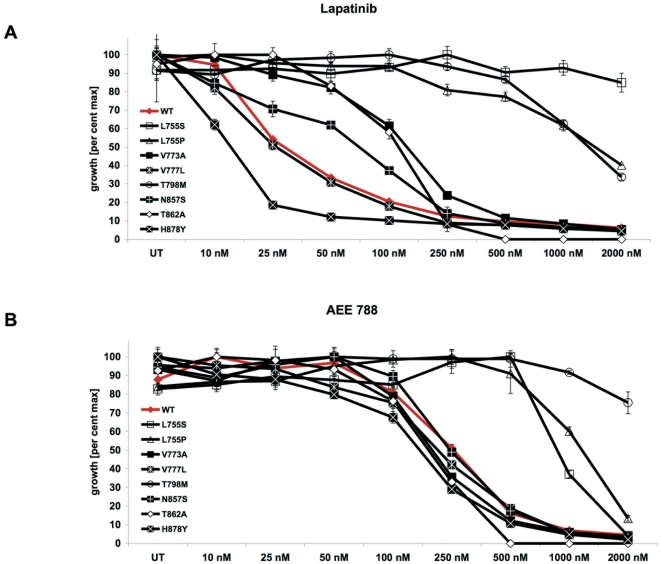
Analysis of ERBB2 kinase domain mutants identifies lapatinib-resistant mutations. Ba/F3 cells stably expressing either wild type or mutant ERBB2 were treated with indicated concentrations of either lapatinib (A) or AEE 788 (B) for 48 hours and analyzed for cell proliferation inhibition.

We next aimed to establish additional ERBB2 mutant expressing cell lines, which completely depend on the overexpressed ERBB2 for their survival. This allows to study their sensitivity towards different kinase inhibitors in a convenient way. Thus, ERBB2 mutations were cloned into the MiGR1 (MSCV-eGFP) vector and stable expressing Ba/F3 cell lines were established. Both wild type ERBB2 and ERBB2 mutants conferred Ba/F3 cells to cytokine independence (data not shown). We then tested the inhibitory effects of lapatinib (Figure S3) on these stable Ba/F3 cell lines expressing ERBB2 mutants. Cell proliferation analysis showed that the ERBB2-H878Y mutant had the highest sensitivity against lapatinib among all mutations tested with a cellular IC50 value nearly half to that of wild type ERBB2 (Figure 4A and Table 1). A similar sensitizing effect of ERBB2-H878Y towards lapatinib was shown recently in CHO cells measuring autophosphorylation of the receptor [13]. Thus, ERBB2-H878Y, which was reported in 11% of hepatoma patients[5], can be considered as a lapatinib-sensitizing mutation similar to EGFR-L858R that was reported as gefitinib-sensitizing mutation in NSCLC[8]. Another mutation, ERBB2-V777L also remained sensitive to lapatinib with a cellular IC50 value similar to that of wild type ERBB2 (Figure 4A and Table 1). However, all remaining mutations showed a shift towards significant higher cellular IC50 values compared to the wild type receptor (Figure 4A and Table 1). Since levels of up to 1 µM of lapatinib may be achieved in patients, ERBB2-V773A, ERBB2-T862A and ERBB2-N857S (IC90 approximately 0.5 µM) mutations might respond to higher doses of lapatinib. In contrast, ERBB2-L755S (IC50>2 µM), ERBB2-L755P (IC50>1.5 µM) and ERBB2-T798M (IC50>1 µM) caused strong lapatinib resistance (Figure 4A and Table 1). These results indicate that the amino acids L755 and T798 in ERBB2 are critical residues determining lapatinib sensitivity and those patients with these mutations may not respond to lapatinib treatment. In summary, based on lapatinib sensitivity, ERBB2 kinase domain mutations can be classified into three groups: (1) lapatinib-sensitizing (IC50≤30 nM) – ERBB2-H878Y & ERBB2-V777L; (2) lapatinib-sensitive (IC50 value between 30 nM and 1 µM) – ERBB2-V773A, ERBB2-N857S & ERBB2-T862A and (3) lapatinib-resistant (IC50>1 µM) – ERBB2-L755S, ERBB2-L755P & ERBB2-T798M.

Breast cancer patients with wild type ERBB2 kinase may develop secondary resistance to lapatinib due to kinase domain mutations similar to secondary drug resistance reported in NSCLC or CML patients treated with kinase inhibitors. To test the hypothesis whether ERBB2 resistance mutations identified above can lead to secondary drug resistance *in vitro* we performed a classical drug resistance screen as described before using 2 µM of lapatinib (Figure S4). Indeed we were able to recover secondary resistance mutations in this screen (ERBB2-L755S and ERBB2-T862A) indicating the possible emergence of resistance mutations in WT-ERBB2 patients treated with lapatinib (Figure S4). Interestingly, ERBB2-L755S was also reported recently in an *in vitro* lapatinib-resistance screen performed at concentrations 0.4 µM, 0.6 µM, 0.8 µM and 1.2 µM[12]. Thus, comprehensive sequence analysis of secondary lapatinib resistant patients will be necessary in the future to determine whether this is a clinically important resistance mechanism in breast cancer patients as already demonstrated in CML or NSCLC patients.

We next tested whether ERBB2 kinase domain mutations exhibit differential sensitivity towards an alternative reversible ERBB2 inhibitor, AEE788 (Figure S3). Interestingly, overall the efficacy of this inhibitor was not altered by most mutations except ERBB2-L755S, ERBB2-L755P and ERBB2-T798M (Figure 4B and Table 1). While ERBB2-L755S and ERBB2-L755P mutants remained sensitive to AEE788 at very high concentrations (IC90 below 2 µM), the gatekeeper ERBB2-T798M mutation is totally resistant (IC50>2 µM) to AEE788 treatment (Figure 4B). Thus, lapatinib and AEE788 indeed display differential sensitivities to most ERBB2 mutants while ERBB2-L755S, ERBB2-L755P and ERBB2-T798M showed cross-resistance to both inhibitors.

### Structural basis of lapatinib resistance

Structural modeling was performed to elucidate the possible mechanisms for lapatinib resistance due to ERBB2 kinase domain mutations. To date, the crystal structure of ERBB2 has not been solved. However, the high degree of identity and large number of crystal structures available for EGFR makes it well suited to also model structures for the ERBB2 kinase; their ligand binding surfaces at and near the ATP binding site are almost identical (Figure S5).

#### L755S/P

**Figure 5:**
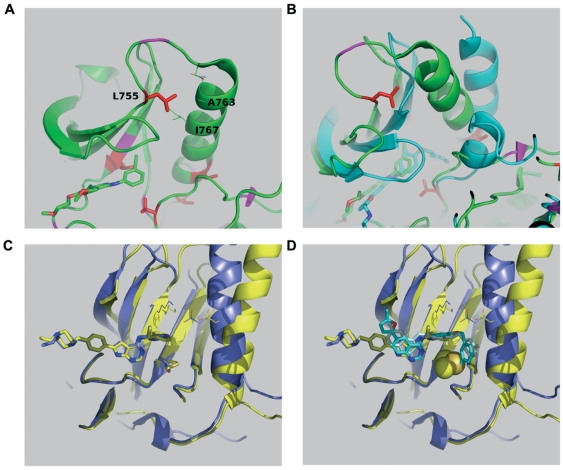
Structural analysis of lapatinib resistant ERBB2 kinase domain mutants. (**A**) L755 packs against helix C, closest to residues Ala763 and Ile767, and makes no contacts with the inhibitors (structure 1M17 with inhibitor erlotinib is depicted lower left). (**B**) Comparing the active structure of 1M17 (green) to an inactive representative 1XKK bound to lapatinib shows the loss of L755 interactions (cyan). (**C**) Overlay of AEE788 bound structures of EGFR (2J6M, active, blue) and EGFR T790M (2JIU, inactive, yellow). The existence of the salt bridge linking the active site lysine K753 with the helix C E770 is a marker for the active state. The T798M (ERBB2 numbering) mutation does not significantly alter binding, although a rotation of the inhibitor aromat is apparent. (**D**) Superposition of two binding modes of lapatinib onto the overlay of figure 2C and display of the T798M atoms as Van der Waals spheres shows how the binding mode seen in 1XKK (cyan) obviously clashes with the mutation, but the binding mode of 3BBT (pale blue, ERBB4, which also has threonine as gatekeeper) does not.

**Figure 6:**
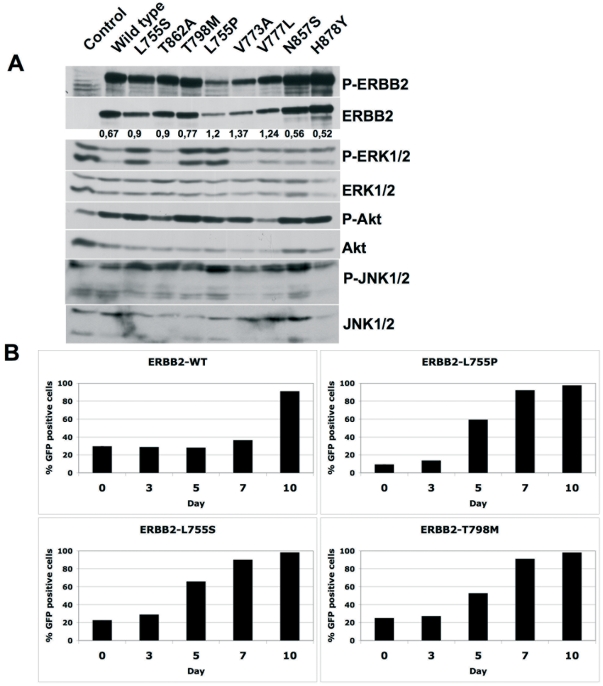
Lapatinib-resistant ERBB2 mutants show increased transformation potential of Ba/F3 cells to cytokine independence. (A) Ba/F3 cells transformed by ERBB2 mutants were analysed by western blotting for the activation of ERBB2 and downstream ERK phosphorylation. (B) To test the activating nature of lapatinib-resistant mutations, Ba/F3 cells were transduced with wild type or mutant MSCV-eGFP-ERBB2 and outgrowth of ERBB2-positive (green) cells with respect to parental (non-green) Ba/F3 cells was measured by FACS analysis at indicated time points.

Figure 5A shows contacts between L755 and helix C that are seen in the active EGFR structures (1M17, 2ITY, 2ITO, 2ITZ, 2ITT, 2TIP). Their geometries are not identical, with three structures showing a significantly displaced position (2ITP, 2ITY, 2ITT) that does not however eliminate the contacts; one of these (2ITY) also shows an additional contact to a displaced aromatic side chain from the glycine loop hairpin aromat F723 (EGFR numbering). While mutations at L755 will not affect inhibitor binding directly, they do affect the packing interactions with helix C, and thus will influence the structure of the active state and the transition between active and inactive forms. In the active form (Figure 5A), L755 packs against the helix with hydrophobic interactions. In inactive forms (Figure 5B), the C-helix is translated away from the active site, the activation loop may adopt a helical turn, and L755 does not make ordered contact with helix C. The activating nature of L755S and L755P mutations is evident from their ability to transform Ba/F3 cells to cytokine independence relatively quickly compared to the wild type ERBB2 kinase in a competition assay (Figure 6B). Moreover, mutations ERBB2-L755S, ERBB2-L755P and ERBB2-T798M showed enhanced MAPK signaling compared to both the wild type and lapatinib-sensitive ERBB2 mutants (Figure 6A). Because the mutations are transforming, the L755S/P mutations either stabilize the active state relative to the inactive state or lower a barrier to activation. L755P may do this by reducing disorder of the inactive state and stabilizing the loop favorable for an active conformation. L755S likely destabilizes the interactions in the inactive state, observed to be hydrophobic. It is also possible that L755S introduces stabilizing polar interactions of a structurally altered active form. In conclusion, mutations affecting L755 seems to stabilize the active conformation of the ERBB2 kinase. This would explain the resistance to lapatinib that targets the inactive conformation of the ERBB2 kinase and the partly retained sensitivity to AEE778 that target preferentially the active conformation[14].

#### T798M

Threonine 798 is the ERBB2 “gatekeeper”, the ATP site residue long known as a primary selectivity determinant among protein kinases. The gatekeeper is also known as the most prominent site of drug resistant mutations of Abl kinase against imatinib and other CML drugs. In these cases, the mutation is T->I, which is transforming of itself and also lowers drug binding strengths[15]. The mutation of the gatekeeper threonine to methionine is the principle mechanism for drug resistance in EGFR kinase (T790M). It is known to enhance the affinity of oncogenic forms of EGFR kinase to ATP[16], explaining its drug resistant properties despite retention of the ability to bind EGFR inhibitors. In line with this assumption ERBB2-T798M displays increased transforming potential compared to wild type ERBB2 (Figure 6B). Figure 5C shows how the binding mode of AEE788 remains unaffected by the ERBB2-T798M mutation. Thus, the increased affinity of ERBB2-T798M towards ATP might explain the observed inhibitor resistance towards the reversible inhibitor AEE788. Figure 5D shows different binding modes for lapatinib in EGFR kinase and ERBB4, which share high identity with ERBB2. The binding mode as modelled in EGFR kinase is not compatible with the T798 mutation, although the binding mode seen in ERBB4 may be so. Moreover, unlike AEE788, lapatinib binds the inactive conformation preferentially. Thus, the stabilization of an active conformation in ERBB2-T798M in combination with increased affinity to ATP might contribute to lapatinib resistance.

### Irreversible inhibitors potently inhibits drug resistant ERBB2 mutants

**Figure 7:**
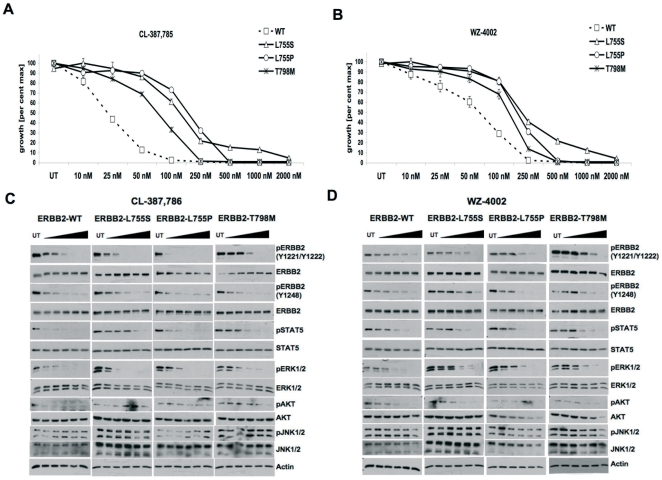
Irreversible inhibitors overcome lapatinib resistance due to ERBB2 kinase domain mutations. Stable Ba/F3 cell lines expressing either wild type or mutant ERBB2 were treated with indicated concentrations of either CL-387785 (**A**) or WZ-4002 (**B**) for 48 hours and analyzed for inhibibtion of cell proliferation. Indicated Ba/F3-ERBB2 cell lines were treated with increasing concentrations (50 nM, 100 nM, 250 nM, 500 nM or 1000 nM) of either CL-387785 (**C**) or WZ-4002 (**D**) for 30 minutes and analyzed by western blotting with indicated antibodies.

CL-387785 is an irreversible EGFR/ERBB2 inhibitor that was shown to overcome gefitinib resistance due to the EGFR-T790M gatekeeper mutation[17]. WZ-4002 was recently reported to have significant in vitro and in vivo activity against both the wild type and mutant EGFR[18], [19]. Moreover, irreversible inhibitors were recently shown to overcome inhibitor resistance caused due to insertion mutations in the ERBB2 kinase[20]–[22]. Thus, we tested the efficacy of these irreversible inhibitors CL-387785 and WZ-4002 (Figure S3) on lapatinib-resistant ERBB2 point mutations (L755S, L755P and T798M). Interestingly, both inhibitors potently inhibited proliferation of Ba/F3-ERBB2 mutant cell lines with IC50 values less than 200 nM (Figure 7A and 7B). WZ-4002 was more potent (fold-increase of IC50 of mutant ERBB2 compared to wild type ERBB2) than CL-387785 (Table S2). Biochemical analysis of ERBB2 kinase activity and downstream targets showed that both irreversible inhibitors showed significant activity towards all three resistant ERBB2 mutants (Figure 7C and 7D). The structural basis for the excellent activity of WZ-4002 against lapatinib resistant ERBB2 mutations may be attributed to its ability to bind an active conformation of the ERBB2 kinase in an irreversible manner. Thus, WZ-4002 may be a potential alternative compound to treat cancer patients with either primary or secondary lapatinib resistance due to ERBB2 kinase domain mutations located at L755 or T798 within a clinical trial.

In summary, in this study lapatinib-resistant ERBB2 kinase domain mutations were identified and the efficacy of irreversible inhibitors to overcome lapatinib resistance is demonstrated. Moreover, an ERBB2 mutant (H878Y) observed in 11% of hepatocellular carcinoma patients showed remarkable sensitivity to lapatinib indicating that lapatinib may be an attractive option in the future for hepatoma patients with ERBB2-H878Y.

## Materials and Methods

### Chemical reagents, DNA constructs and cell culture

Erlotinib and lapatinib was purchased from the pharmacy. Gefitinib was kindly provided by AstraZeneca, and AEE788 was a kind gift from Novartis Pharma AG, Basel. CL-387785 was purchased from Calbiochem and WZ-4002 was purchased from Axon Medchem. Each compound was dissolved in DMSO to make an initial stock solution of 10 mmol/L (gefitinib, AEE788 and WZ-4002), 2.5 mmol/L (erlotinib and lapatinib) and 1 mmol/L (CL-387785). Human EGF was purchased from Chemicon and recombinant human Heregulin was purchased from Calbiochem.

MiGR1-ERBB2 and pcDNA-ERBB3 were a kind gift from Prof. Dr. Helga Bernhard. Point mutations were introduced in to MiGR1-ERBB2 as described previously[9], [10]. All mutations were confirmed by sequencing.

Ba/F3 cells were cultured in RPMI 1640 (Life Technologies) supplemented with 10% FCS, glutamine, and interleukin-3 (IL-3; R&D Systems). Stable Ba/F3 cell lines expressing wild type or mutant ERBB2 were established by retroviral infection with MiGR1-ERBB2 followed by IL-3 withdrawal. HEK293 cells were cultured in DMEM (Life Technologies) supplemented with 10% FCS. Murine mammary epithelial cell line NMuMg was cultured in DMEM supplemented with 10% FCS, NaHCO_3_ and insulin. Stable NMuMg cell lines were established by retroviral infection with either wild type or mutant ERBB2 constructs.

### Western blotting, soft agar assay, and cell proliferation assay

HEK293 cells were transfected with MiGR1-ERBB2 constructs either alone or in combination with EGFR/ERBB3 cDNA for 36 hours before serum starvation for 12 hours. Cells were then stimulated with either 25 ng/ml of human EGF (Chemicon) or 50 ng/ml of heregulin (Calbiochem) for 5 minutes and pelleted for cell lysis. Ba/F3 cells[9] expressing either wild type or mutant ERBB2 constructs were treated with either CL-387785 or WZ-4002 for 30 minutes and pelleted. Cell lysis, SDS-PAGE and Western blotting were done as described previously[9], [10]. The following antibodies were used: phosphorylated ERBB2-Tyr^1248^ (Millipore), ERBB2-Tyr^1221/1222^ (Santa Cruz Biotechnology), ERBB2 (Santa Cruz Biotechnology), p44/42 mitogen-activated protein kinase [extracellular signal-regulated kinase (ERK1/ERK2)] (Cell Signaling), phosphospecific ERK1/ERK2 (Cell Signaling), pStat5-Tyr^694^ (Cell Signaling), Stat5 (Santa Cruz Biotechnology), p-SAPK/JNK (Thr183/Tyr185) (Cell Signaling), SAPK/JNK (Cell Signaling), pAKT (Ser473) (Cell Signaling), and AKT1/2 (Santa Cruz Biotechnology). Bands were visualized using the enhanced chemiluminescence system (Amersham).

Anchorage-independent cell growth was analysed by colony formation ability in soft agar assay as described previously[19]. Analysis of cell proliferation was done using an 3-(4,5-dimethylthiazol-2-yl)-5-[3-carboxymethoxyphenyl-2-(4-sulfophenyl)-2H-tetrazolium (MTS)]-based method by absorption of formazan at 490 nm (CellTiter 96; Promega). Samples were measured in triplicates after 48 h of culture in indicated drug concentrations.

### Lapatinib resistance screen

Ba/F3 cells stably expressing wild type ErbB2 were treated twice with 100 µg/mL of N-ethyl-N-nitrosourea (ENU) for 12 hours. Cells were then washed thoroughly and cultured in 96-well plates at a density of 4×10^5^ per well in the presence of 2 µM lapatinib. Lapatinib resistant cell colonies were isolated. Total RNA was extracted using TRIzol reagent (Invitrogen). cDNA encompassing ErbB2 kinase domain was synthesized by one step reverse-transcription PCR (Promega) and sequenced.

### Structural analysis of lapatinib resistant ERBB2 mutants

Crystal structure coordinates for inhibitor complexes with the ErbB1 kinase domain (ErbB1-KD), ErbB1-KD mutations, and ErbB4-KD are available from the Protein Data Bank (www.pdb.org). Crystal structures of complexes with erlotinib (1M17), lapatinib (1XKK, 3BBT), gefitinib (2ITY, 2ITO, 2ITZ), and AEE788 (2J6M, 2ITP, 2ITT, 2JIU), representing both active and inactive states of the kinase domain, were superimposed and inspected using the graphics program PyMOL (www.pymol.org)[14], [16], [23]–[25].

## Supporting Information

Figure S1 **Colony formation by early-passage NMuMg cells stably expressing ERBB2 mutants.** 2.5×10^4^ cells per well were plated in a six-well plate and analyzed for colony formation. NMuMg cell line infected with MiGR1 vector is used as a control.
(TIF)

Figure S2 **Colony formation by late-passage NMuMg cells.** Late-passage NMuMg cells stably expressing ERBB2 mutants were analyzed for colony formation. Cells infected with MiGR1 vector is shown as control.
(TIF)

Figure S3 **Structures of reversible (A) and irreversible (B) inhibitors used in this study.**
(TIF)

Figure S4 **Cell-based screen for lapatinib resistance.** Schematic representation of lapatinb resistance screen performed with Ba/F3 cells stably expressing wild type ERBB2 kinase **(A)**. Residues affected by lapatinib resistance mutations identified in the *in vitro* screen were conserved in other ERBB members except ERBB3 **(B)**.
(TIF)

Figure S5 **Surface representation of EGFR kinase.** Surface representation of EGFR (in complex with erlotinib, 1M17), showing potential binding surfaces attributable to residues that differ between EGFR and ERBB2. Only one site is within the ATP binding pocket (Cys775->Ser). A second is close by; Phe795 in EGFR is replaced by Tyr803 in ErbB2 visible near an ether chain of the inhibitor to the left of the binding cleft.
(TIF)

Table S1 **Representation of previously reported EGFR mutations homologous to ERBB2 mutants that were analyzed in this study.**
(TIF)

Table S2 **Summary of relative resistance profiles of ERBB2 mutants against AEE 788, CL-387785 and WZ-4002 compared to lapatinib.** Approximate fold-increase in IC50 value of indicated ERBB2 mutant compared to wild type ERBB2 are calculated and classified as less (green), moderate (yellow) or highly (red) resistant.
(TIF)
