# Supplementary figures and images for: Differential Sensitivity of ERBB2 Kinase Domain Mutations towards Lapatinib
Source: PLoS One. 2011 Oct 28;6(10):e26760. doi: 10.1371/journal.pone.0026760 (PMC3203921; doi:10.1371/journal.pone.0026760)

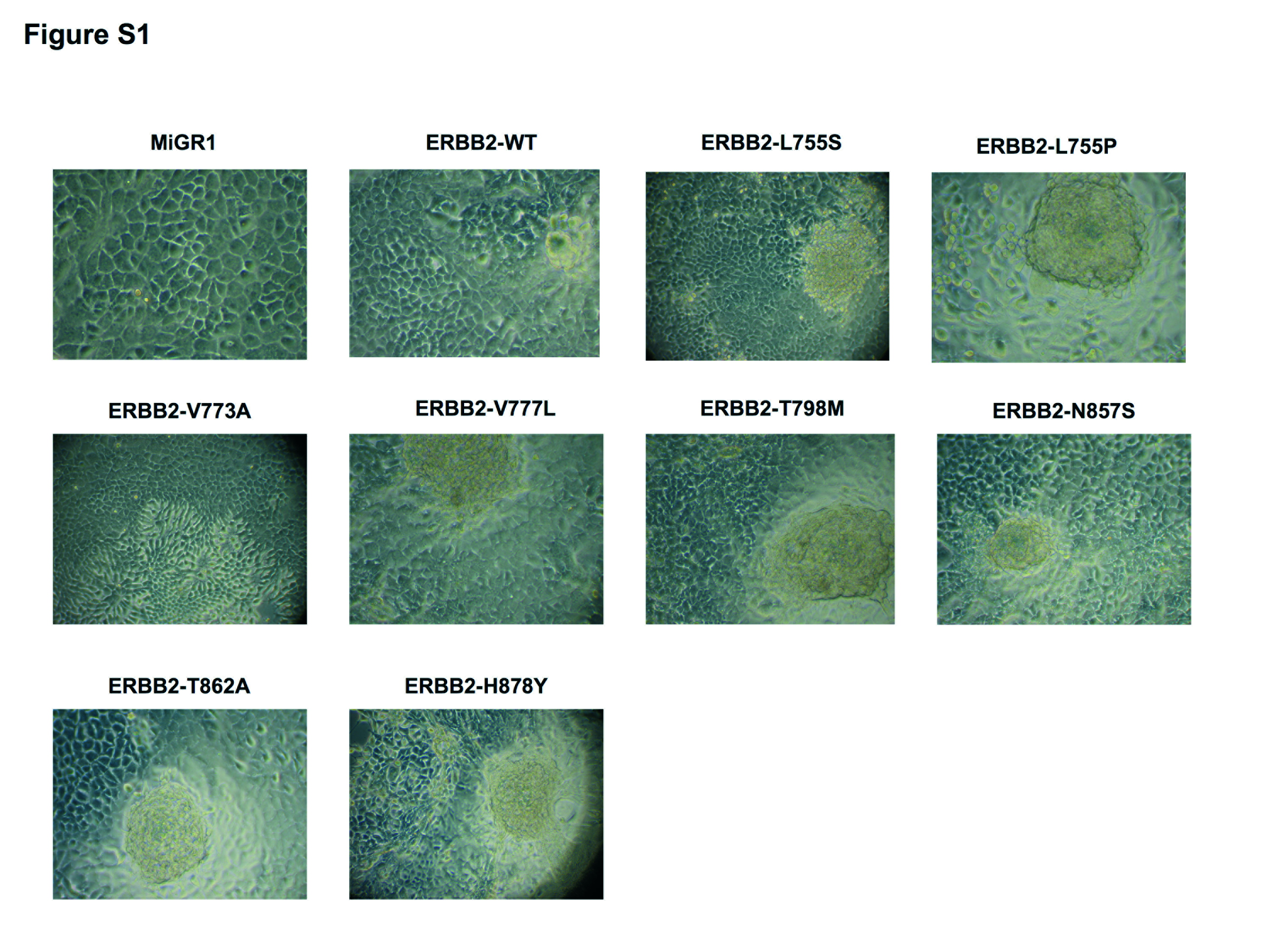

Supplement: Figure S1 — Colony formation by early-passage NMuMg cells stably expressing ERBB2 mutants. 2.5×10^4^ cells per well were plated in a six-well plate and analyzed for colony formation. NMuMg cell line infected with MiGR1 vector is used as a control. (TIF) [file pone.0026760.s001.tif]

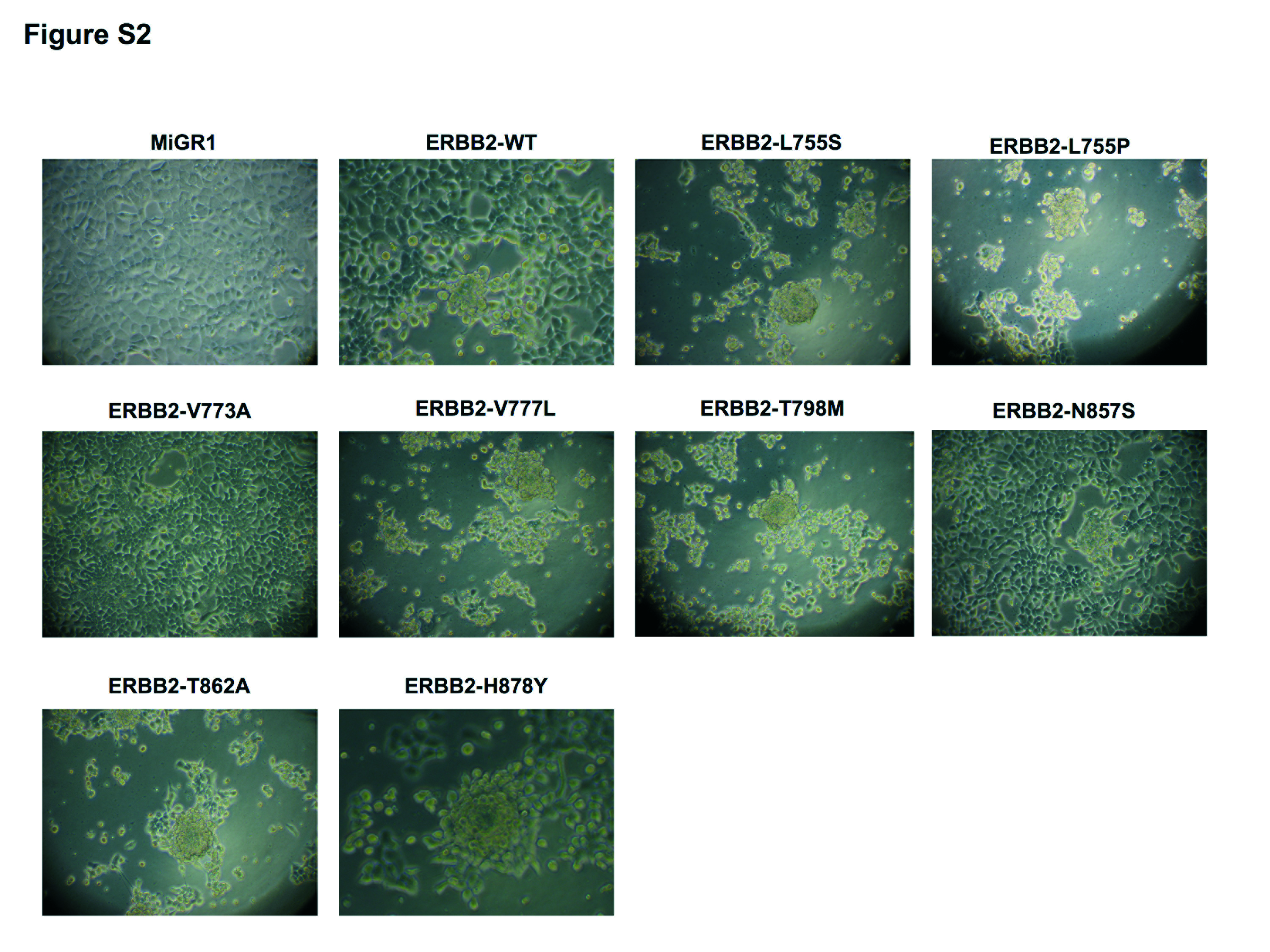

Supplement: Figure S2 — Colony formation by late-passage NMuMg cells. Late-passage NMuMg cells stably expressing ERBB2 mutants were analyzed for colony formation. Cells infected with MiGR1 vector is shown as control. (TIF) [file pone.0026760.s002.tif]

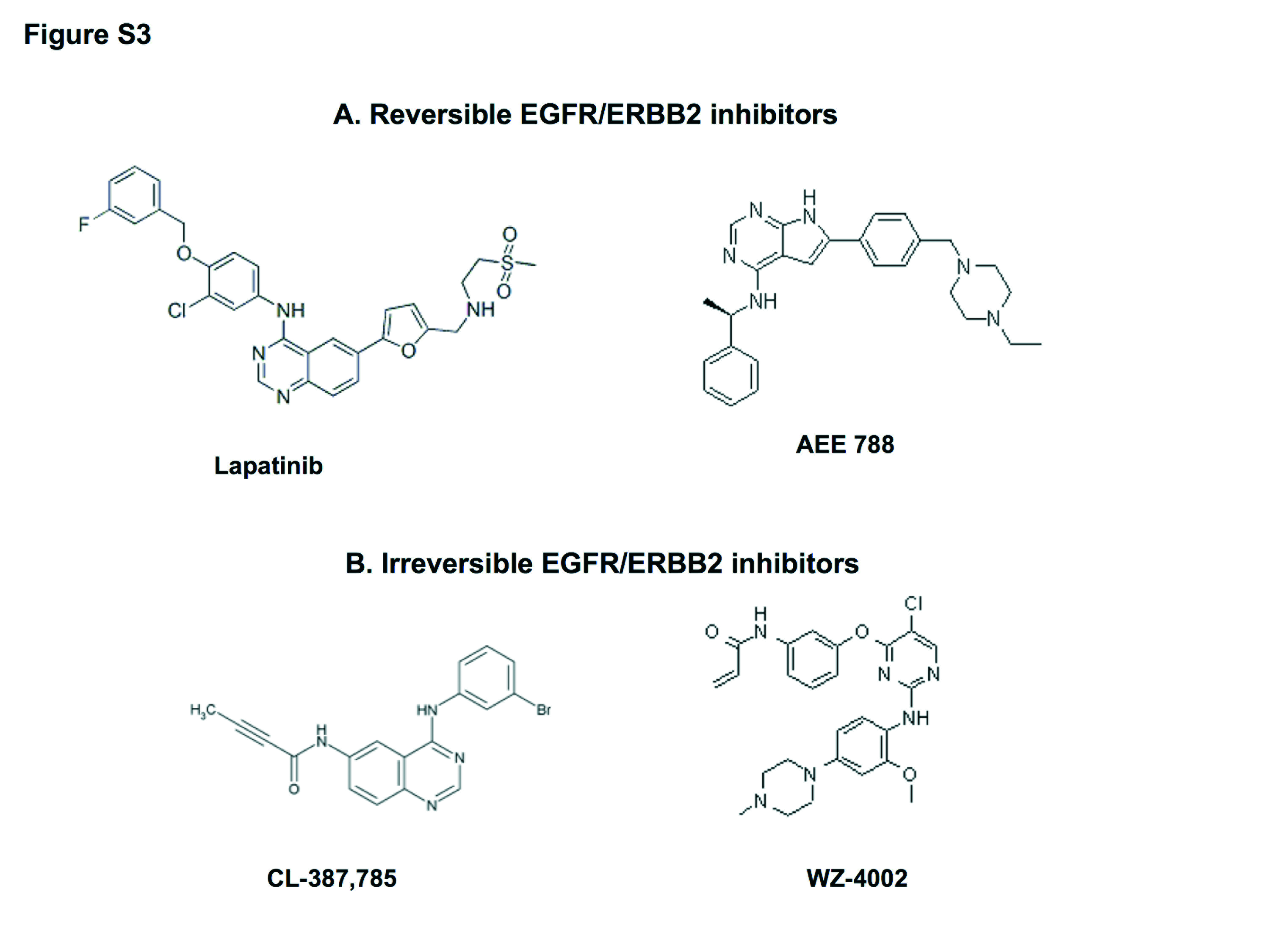

Supplement: Figure S3 — Structures of reversible (A) and irreversible (B) inhibitors used in this study. (TIF) [file pone.0026760.s003.tif]

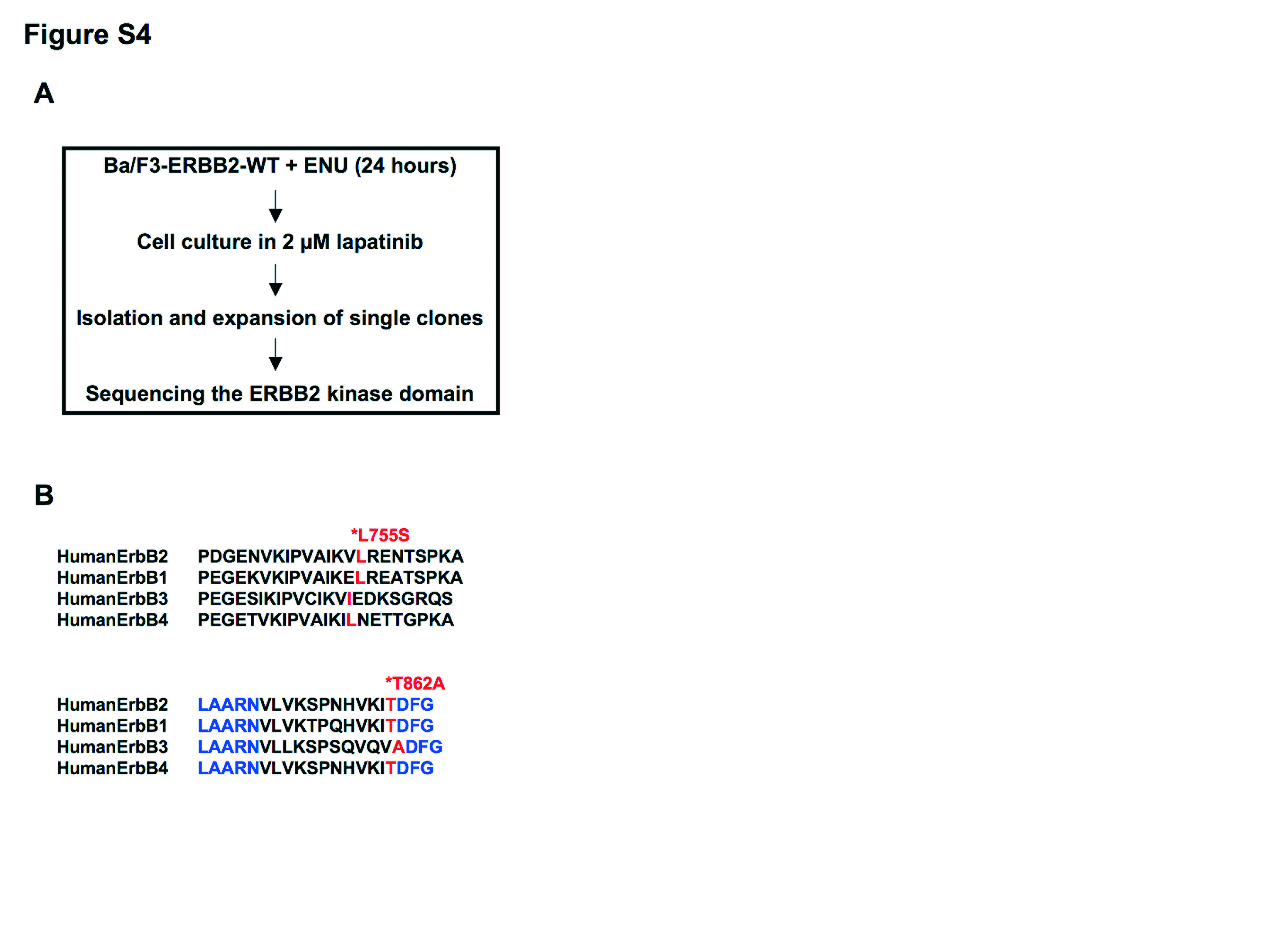

Supplement: Figure S4 — Cell-based screen for lapatinib resistance. Schematic representation of lapatinb resistance screen performed with Ba/F3 cells stably expressing wild type ERBB2 kinase (A). Residues affected by lapatinib resistance mutations identified in the in vitro screen were conserved in other ERBB members except ERBB3 (B). (TIF) [file pone.0026760.s004.tif]

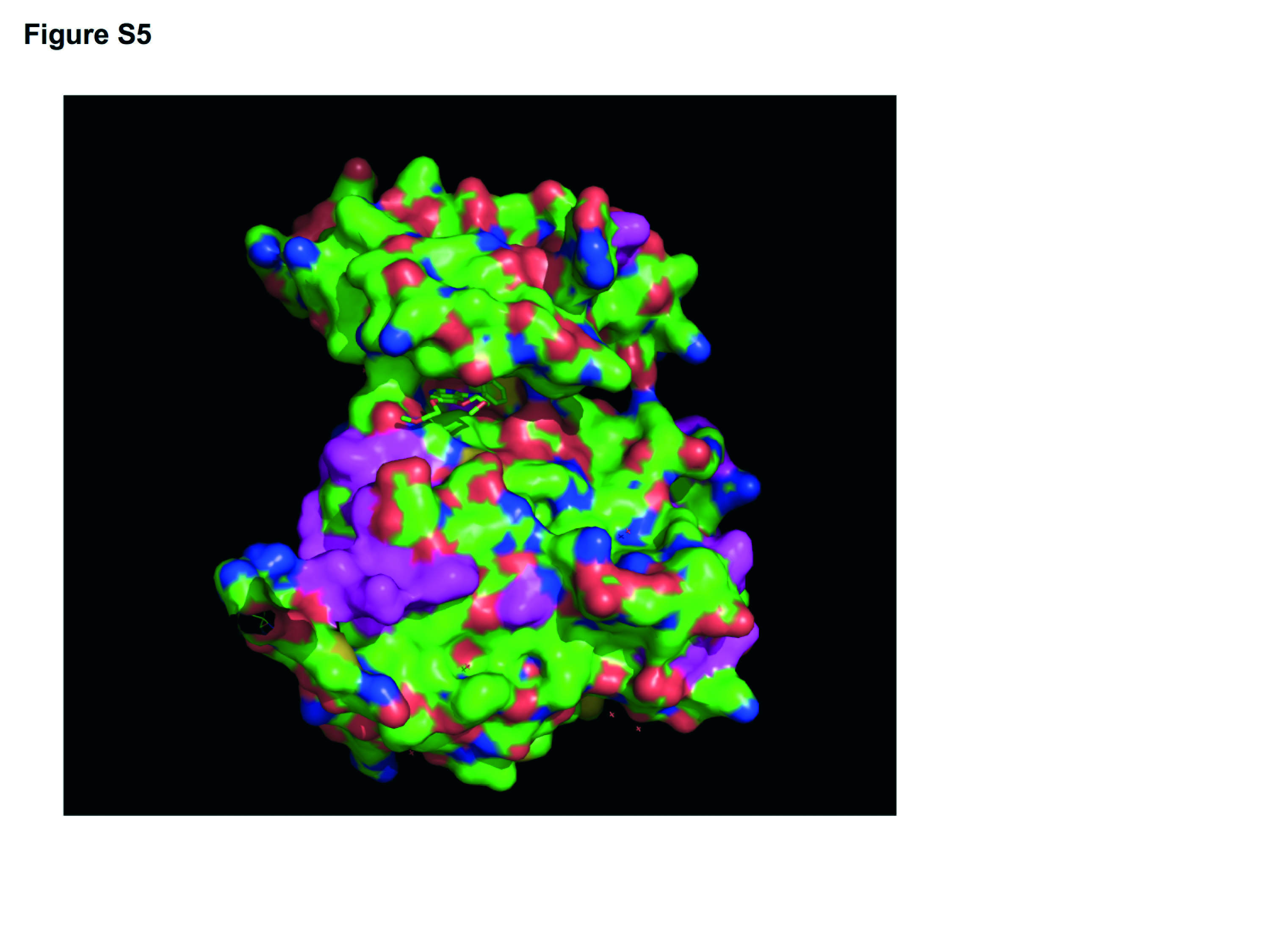

Supplement: Figure S5 — Surface representation of EGFR kinase. Surface representation of EGFR (in complex with erlotinib, 1M17), showing potential binding surfaces attributable to residues that differ between EGFR and ERBB2. Only one site is within the ATP binding pocket (Cys775->Ser). A second is close by; Phe795 in EGFR is replaced by Tyr803 in ErbB2 visible near an ether chain of the inhibitor to the left of the binding cleft. (TIF) [file pone.0026760.s005.tif]

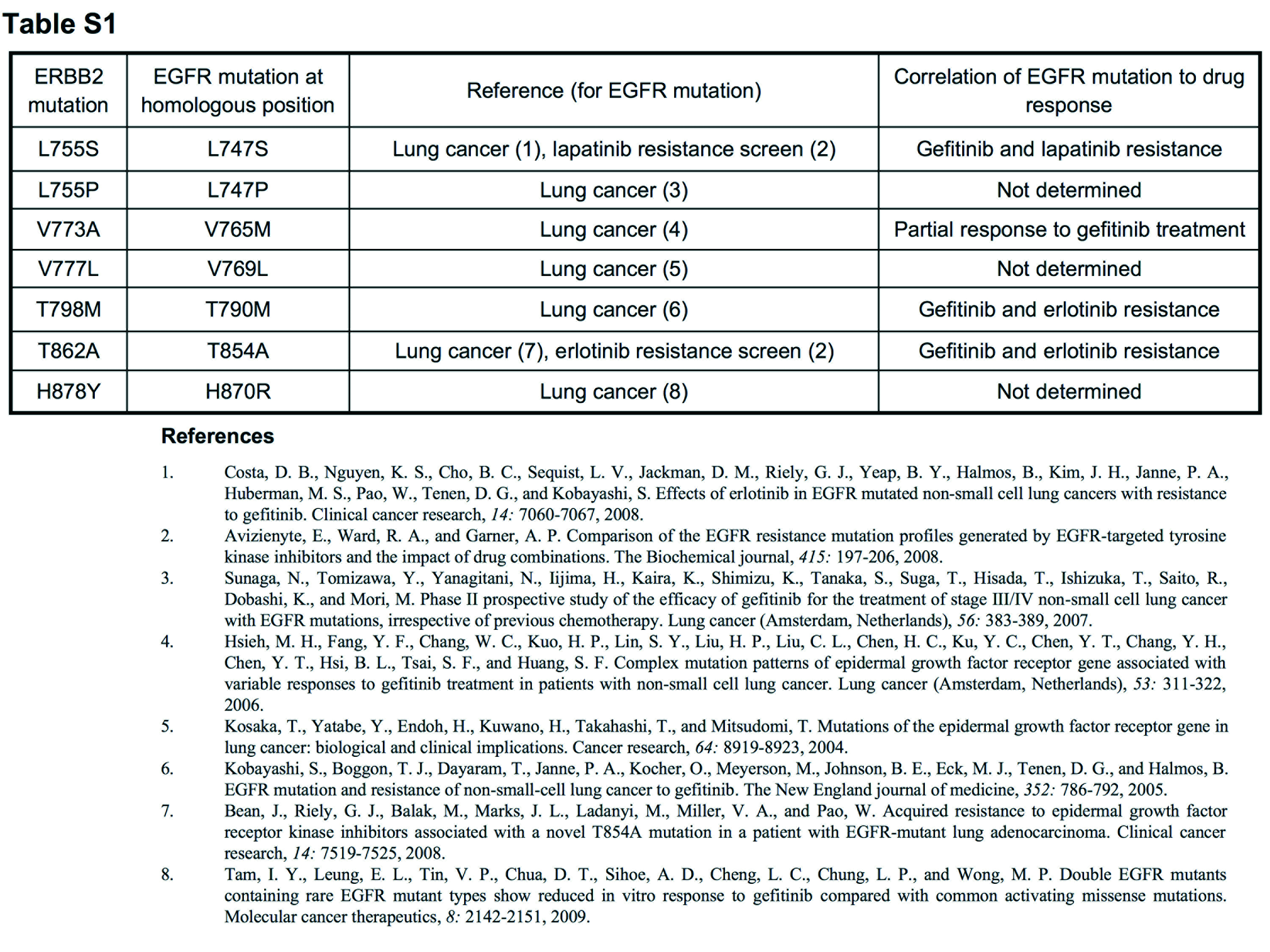

Supplement: Table S1 — Representation of previously reported EGFR mutations homologous to ERBB2 mutants that were analyzed in this study. (TIF) [file pone.0026760.s006.tif]

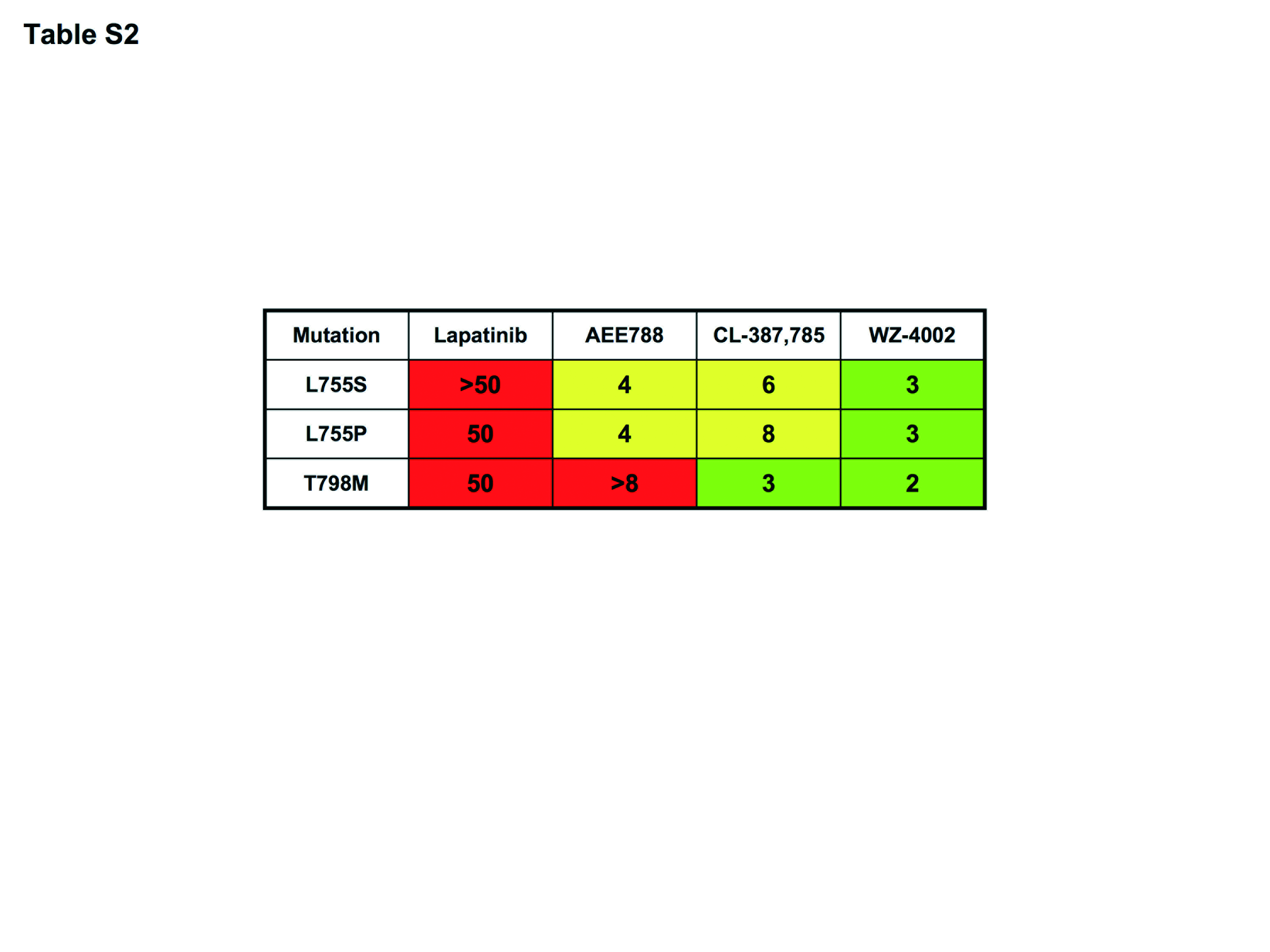

Supplement: Table S2 — Summary of relative resistance profiles of ERBB2 mutants against AEE 788, CL-387785 and WZ-4002 compared to lapatinib. Approximate fold-increase in IC50 value of indicated ERBB2 mutant compared to wild type ERBB2 are calculated and classified as less (green), moderate (yellow) or highly (red) resistant. (TIF) [file pone.0026760.s007.tif]
